# T-cell Responses in Individuals Infected with Zika Virus and in Those Vaccinated Against Dengue Virus

**DOI:** 10.20411/pai.v2i2.188

**Published:** 2017-06-30

**Authors:** Dominic Paquin-Proulx, Fabio E. Leal, Cassia G. Terrassani Silveira, Alvino Maestri, Claudia Brockmeyer, Shannon M. Kitchen, Vinicius D. Cabido, Esper G. Kallas, Douglas F. Nixon

**Affiliations:** 1 Department of Microbiology, Immunology & Tropical Medicine, The George Washington University, Washington, D.C.; 2 Division of Clinical Immunology and Allergy, School of Medicine, University of São Paulo, São Paulo, SP, Brazil

**Keywords:** zika virus, dengue virus, T cell, IFNγ, ELISPOT, CD4, antibody dependent enhancement

## Abstract

**Background:**

The outbreak of Zika virus (ZIKV) infection in Brazil has raised concerns that infection during pregnancy could cause microcephaly and other severe neurodevelopmental malformations in the fetus. The mechanisms by which ZIKV causes fetal abnormalities are largely unknown. The importance of pre-infection with dengue virus (DENV), or other *flaviviruses* endemic to Brazil, remains to be investigated. It has been reported that antibodies directed against DENV can increase ZIKV infectivity by antibody dependent enhancement (ADE), suggesting that a history of prior DENV infection might worsen the outcome of ZIKV infection.

**Methods:**

We used bioinformatics tools to design 18 peptides from the ZIKV envelope containing predicted HLA-I T-cell epitopes and investigated T-cell cross-reactivity between ZIKV-infected individuals and DENV-vaccinated subjects by IFNγ ELISPOT.

**Results:**

Three peptides induced IFNγ production in both ZIKV-infected subjects and in DENV-vaccinated individuals. Flow cytometry indicated that 1 ZIKV peptide induced a CD4+ T-cell response in DENV-vaccinated subjects.

**Conclusions:**

We demonstrated that vaccination against DENV induced a T-cell response against ZIKV and identified one such CD4+ T-cell epitope. The ZIKV-reactive CD4+ T cells induced by DENV vaccination and identified in this study could contribute to the appearance of cross-reactive antibodies mediating ADE.

## INTRODUCTION

Zika virus (ZIKV) infection was first identified in the Ziika forest of Uganda in 1947 [1]. This virus is a member of the *Flaviviridae,* a family of viruses that also includes Yellow Fever (YF), West Nile, and Dengue viruses (DENV), and ZIKV can be transmitted via mosquito bites, through sexual contact or blood transfusions, or from an infected pregnant woman to her developing fetus. Until recently, ZIKV was understudied because the infection was thought to be associated only with a mild viral illness of limited duration. It is estimated that only 20% of infected humans show signs of ZIKV infection during the acute phase, with symptoms including skin rash, headache, asthenia, and conjunctivitis. In 2014, the virus suddenly expanded its range dramatically and appeared in the Americas, leading to the most widespread ZIKV outbreak recorded. The recent epidemic of ZIKV in Brazil has raised concerns that ZIKV infection during pregnancy could cause severe neurodevelopmental malformations in the fetus, including microcephaly [2, 3]. Evidence includes epidemiological studies showing a strong temporal and geographical association between ZIKV and microcephaly[4], detection of ZIKV in the brain of infants born with microcephaly [3, 5, 6], and ZIKV neurological damage in animal models [7–9]. Three different studies performed in French Polynesia (retrospective), Rio de Janeiro, and Bahia have estimated the risk of microcephaly to be between 0.95% and 29% for pregnant women infected with ZIKV [10–13]. A more recent study in the United States estimated the risk to be 6%, with an increased vulnerability if ZIKV infection occurred during the first trimester [14]. A temporal association between an increase in the incidence of microcephaly cases and ZIKV infection was also noted in Colombia [15]. The mechanisms by which ZIKV causes fetal abnormalities are largely unknown. In fact, we still do not know the risk factors associated with fetal microcephaly among pregnant women.

The importance of pre-infection with DENV, another *flavivirus* endemic to Brazil, remains to be investigated. It has been reported that antibodies directed against DENV can increase ZIKV infectivity *in vitro* by antibody dependent enhancement (ADE) [16–19], suggesting that a history of prior DENV infection might worsen the outcome of a subsequent ZIKV infection. However, the clinical relevance of ADE to ZIKV pathology remains to be determined [20]. Several studies have suggested the possible involvement of cross-reactive T-cell epitopes in DENV pathogenesis. More specifically, memory T-cell clones generated during primary dengue infection in response to epitopes from 1 dengue serotype can cross-react with epitope variants presented during a subsequent infection with a different dengue serotype. We have previously shown differential serotype-specific CD8+ T-cell immunogenicity of DENV proteins [21]. In mice previously infected with DENV, the CD8 T-cell response to ZIKV infection was almost exclusively driven by a limited number of ZIKV/DENV cross-reactive memory cells [22]. To date, no ZIKV or ZIKV/DENV cross-reactive epitopes have been identified in humans. Here, we hypothesized that prior DENV vaccination impacts immunity to ZIKV through T-cell cross-reactivity.

In this study, we investigated virus specific T-cell cross-reactivity between ZIKV and DENV. We selected 18 peptides from the ZIKV envelope with predicted epitopes for the most common HLA-I alleles in Brazil, based on their sequence similarity/dissimilarity with DENV. We identified 3 peptides that induced an IFNγ response in ZIKV-infected patients and in DENV-vaccinated individuals. One of these peptides was found to be a CD4+ T-cell epitope.

## MATERIALS AND METHODS

### Human participants

We selected 7 ZIKV-infected and 9 DENV-vaccinated individuals from São Paulo, Brazil. All participants were enrolled after signing a written consent form approved by the University of São Paulo's Institutional Review Board (CAPPesq 0652/09 and 553/08, respectively). Samples from the DENV-vaccinated individuals were collected between January and August 2014 before the ZIKV outbreak reached São Paulo and selected based on the expression of at least 1 allele of the following HLA-I: HLA-A2, HLA-A24, and HLA-B44. Of the ZIKV-infected patients, 2 reported previous DENV infection (801 and 533). None of the DENV-vaccinated individuals reported previous DENV infection. Blood from control subjects was obtained from the New York Blood Bank. Peripheral blood mononuclear cells (PBMCs) were isolated by density-gradient sedimentation using Ficoll-Paque (Lymphoprep, Nycomed Pharma, Oslo, Norway). Isolated PBMCs were washed twice in Hank's balanced salt solution (Gibco, Grand Island, NY) and cryopreserved in RPMI 1640 (Gibco), supplemented with 20% heat-inactivated fetal bovine serum (FBS; Hyclone Laboratories, Logan UT), 50 U/ml of penicillin (Gibco), 50 μg/ml of streptomycin (Gibco), 10mM glutamine (Gibco) and 10% dimethylsulfoxide (DMSO; Sigma, St Louis, MO). Cryopreserved cells from all participants were stored in liquid nitrogen until used in the assays.

#### DNA extraction and HLA typing

DNA was extracted from peripheral blood using Qiagen kits (QIAamp DNA Mini Kit, Qiagen, Inc, Valencia, CA). The HLA A, B, C and DRB1 typing was performed using PCR and amplification using Sequence-Specific Oligonucleotide (SSP) contained in LABType kits (One Lambda, Inc, Canoga Park, CA).

### Epitope prediction and sequence alignment

The HLA-I epitope predictions were made using Net CTL 1.2 [23] (with a prediction score threshold for epitope identification of 0.75), and HLA-II binding predictions were made using the IEDB analysis resource consensus tool [24, 25]. Amino acid sequences were aligned using the EMBL-EBI bioinformatics framework [26, 27]. Sequence data from Zika MR766 (NCBI Reference Sequence: YP_002790881.1), DENV-1 (UniProtKB/Swiss-Prot: Q1L6I3), DENV-2 (Uni-ProtKB/Swiss-Prot: B8QED9), DENV-3 (UniProtKB/Swiss-Prot: Q2YH64), and DENV-4 (Uni-ProtKB/Swiss-Prot: Q5UU53) were used.

### ELISPOT

Cryopreserved PBMC specimens were thawed, washed, and the cell viability was assessed using the Countess Automated Cell Counter system (Invitrogen, Carlsbad, CA). IFNγ ELISPOT assays were conducted following the manufacturer's instructions (Mabtech, Cincinnati, OH). The ZIKV peptides (GenScript, Piscataway, NJ) were reconstituted with DMSO or water at a concentration of 1 mg/ml and used at a final concentration of 10 μg/mL in duplicate. The PBMCs were plated at a concentration of 1 × 10^5^ cells per well. Spots were counted using AID EliSpot Reader software v6.0. Results from unstimulated PBMCs were used to determine the background level of IFNγ production and multiplied by 2 for each donor individually.

### Flow cytometry and monoclonal antibodies

Freshly thawed PBMCs were labeled with CellTrace Violet (Invitrogen) following the manufacturer's instructions and cultured for 6 days at 37°C and 5% CO_2_ in RPMI medium supplemented with 10% fetal bovine serum (FBS) and 20 units/ml of IL-2 (Sigma), in the presence or absence of 10 μg/ml of peptide. The PBMCs were then re-stimulated with peptide in the presence of monensin (Golgi Stop, BD Biosciences, San Jose, CA) for 12 hours. Cells were washed and stained in Brilliant Violet Stain Buffer (BD Biosciences) at room temperature for 15 minutes in 96-well V-bottom plates in the dark. Samples were then washed and fixed using Cytofix/Cytoperm (BD Biosciences) before flow cytometry analysis. Intracellular staining was performed in Perm/Wash (BD Biosciences), and the following mAbs were used, CD3 PerCP-Cy5.5 (clone UCHT1), CD4 BV605 (clone RPA-T4), CD8 BV711 (clone RPA-T8), and IFNγ APC (clone B27), all from BD Biosciences. Live/dead Fixable Violet Cell Stain was from Life Technologies (Eugene, OR). All data was acquired on a BD LSRFortessa X-20 instrument (BD Biosciences) and analyzed using FlowJo Version 9.9.4 software (TreeStar, Ashland, OR).

## RESULTS AND DISCUSSION

**Figure 1.**
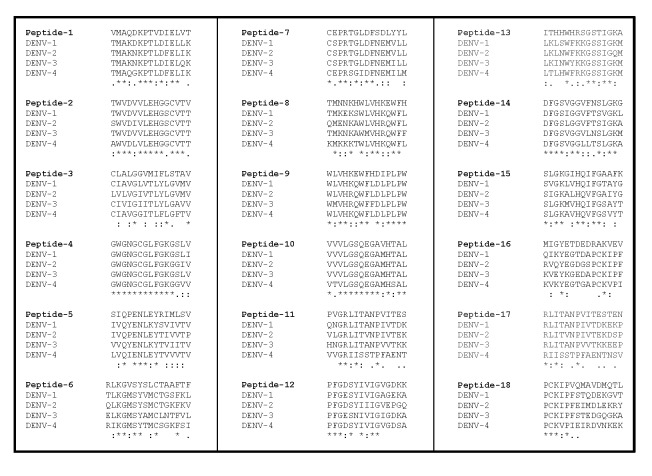
**Alignment of ZIKV and DENV Sequences.** The ZIKV amino acid sequences of selected peptides were aligned to the corresponding DENV-1, DENV-2, DENV-3, and DENV-4 sequences using EMBL-EBI bioinformatics framework. * indicates positions with a single, fully conserved residue;: indicates conservation between groups of strongly similar properties—roughly equivalent to scoring > 0.5 in the Gonnet PAM 250 matrix;. indicates conservation between groups of weakly similar properties—roughly equivalent to scoring ≤ 0.5 and > 0 in the Gonnet PAM 250 matrix.

First, we used Net CTL 1.2 [23] with a threshold of 0.75 to predict the HLA-I epitopes from the ZIKV envelope for 3 of the most common alleles in Brazil (HLA-A2 approximately 25%, HLA-A24 approximately 10%, and HLA-B44 approximately 11%) [28, 29]. We identified between 9 and 11 partially overlapping 9-mers for each allele (Table 1). We then proceeded to map these epitopes on an alignment of the ZIKV envelope with the envelopes of DENV 1-4 in UniProt [30]. Based on the similarity of the Net CTL-predicted epitopes between these viruses, we designed 18 peptides (17 peptides were 15-mers and one was a 16-mer) containing at least 1 predicted epitope each (Table 2). Some of the ZIKV peptides had a high similarity with all DENV serotypes (peptides 1, 2, 4, 9, 10, and 14), and some others had a much lower similarity (peptides 3, 16, 17, and 18) (Figure 1).

**Table 1.** NetCTL Predicted Epitopes

	peptide sequence	Predicted HLA-I binding affinity	C terminal cleavage affinity	TAP transport efficiency	Prediction score
HLA-A2	VMAQDKPTV	0.6314	0.7253	0.5250	1.0763
	GLFGKGSLV	0.5499	0.9620	0.2600	0.9770
	TMNNKHWLV	0.6882	0.9214	0.3690	1.1826
	RLKGVSYSL	0.4368	0.9783	1.2640	0.8611
	QMAVDMQTL	0.4571	0.7170	1.1530	0.8466
	RLITANPVI	0.6463	0.8378	0.8370	1.1310
	SLGKGIHQI	0.5049	0.9345	0.5850	0.9220
	GMSWFSQIL	0.5587	0.9007	0.8630	1.0111
	QILIGTLLV	0.5145	0.9302	0.5280	0.9328
	ALGGVMIFL	0.6719	0.8577	1.0030	1.1805
	VMIFLSTAV	0.7082	0.9511	0.5370	1.2252
HLA-A24	QYVCKRTLV	0.2878	0.8548	0.6800	0.7752
	GWGNGCGLF	0.2630	0.4394	2.5710	0.7544
	HWLVHKEWF	0.4919	0.0586	2.8090	1.1966
	WFHDIPLPW	0.3512	0.7479	0.8880	0.9376
	SYSLCTAAF	0.7168	0.8959	2.8950	1.8055
	SLCTAAFTF	0.3226	0.8830	2.5640	0.9477
	PFGDSYIVI	0.3516	0.3935	-0.0860	0.8034
	HWHRSGSTI	0.5154	0.6547	0.6970	1.2305
	DFGSVGGV	0.3936	0.4993	2.1670	1.0215
HLA-B44	LEHGGCVTV	0.5892	0.9765	0.2600	1.6195
	PENLEYRIM	0.3196	0.2960	-0.4540	0.8138
	LEYRIMLSV	0.5119	0.9701	0.5110	1.4396
	DEDRAKVEV	0.2847	0.7879	-0.3030	0.8086
	CEPRTGLDF	0.2820	0.1832	2.2040	0.8364
	LDFSDLYYL	0.2548	0.7789	0.7330	0.7849
	KEWFHDIPL	0.7230	0.8664	1.0750	1.9754
	QEGAVHTAL	0.6119	0.9682	0.7230	1.6977
	SQILIGTLL	0.3871	0.4819	1.0470	1.0839

**Table 2.** Selected ZIKV Peptides

Peptide number	Sequence	Position in envelope	Predicted MCH-I allele
1	**VMAQDKPTV**DIELVT	33-47	A2
2	TWVDVV**LEHGGCVTV**	19-33	B44
3	CLALGG**VMIFLSTAV**	484-498	A2
3	CL**ALGGVMIFL**STAV	484-498	A2
4	GWGNGC**GLFGKGSLV**	100-114	A2
4	**GWGNGCGLF**GKGSLV	100-114	A24
5	SIQ**PENLEYRIM**LSV	129-143	B44
5	SIQPEN**LEYRIMLSV**	129-143	B44
6	**RLKGVSYSL**CTAAFTF	295-310	A2
6	RLKGV**SYSLCTAAFTF**	295-310	A24
6	RLKGVSY**SLCTAAFTF**	295-310	A24
7	**CEPRTGLD**FSDLYYL	186-200	B44
7	CEPRTG**LDFSDLYYL**	186-200	B44
8	**TMNNKHWLV**HKEWFH	201-215	A2
8	TMNNK**HWLVHKEWF**H	201-215	A24
9	WLVH**KEWFHDIPL**PW	207-221	B44
9	**WLVHKEW**FHDIPLPW	207-221	A24
9	WLVHKE**WFHDIPLPW**	207-221	A24
10	VVVLGS**QEGAVHTAL**	251-265	B44
11	PVGR**LITANPVITES**	350-365	A2
12	**PFGDSYIVI**GVGDKK	377-391	A24
13	ITH**HWHRSGSTI**GKA	392-406	A24
14	**DFGSVGGV**FNSLGKG	426-440	A24
15	**SLGKGIHQIF**GAAFK	436-450	A2
16	MIGYET**DEDRAKVEV**	151-165	B44
17	**RLITANPVI**TESTEN	353-367	A2
18	PCKIPV**QMAVDMQTL**	334-348	A2
A	**VMAQDKPTV**	33-41	A2
B	QDKPTVDIE	36-44	-
C	PTVDIELVT	39-47	-

Predicted epitopes are indicated in bold.

To validate the specificity of the peptides, we performed ELISPOTS with PBMCs obtained from 10 control patients from an area free of DENV and ZIKV (New York Blood Bank) and stimulated donor cells with each peptide individually. Stimulation with peptide number 16 resulted in IFNγ production above background in 7 out of the 10 donors tested, suggesting that this peptide is cross-reactive with a common antigen. Therefore, we excluded peptide 16 from further analysis. No other peptides induced IFNγ production above background levels in the control PBMCs.

**Figure 2.**
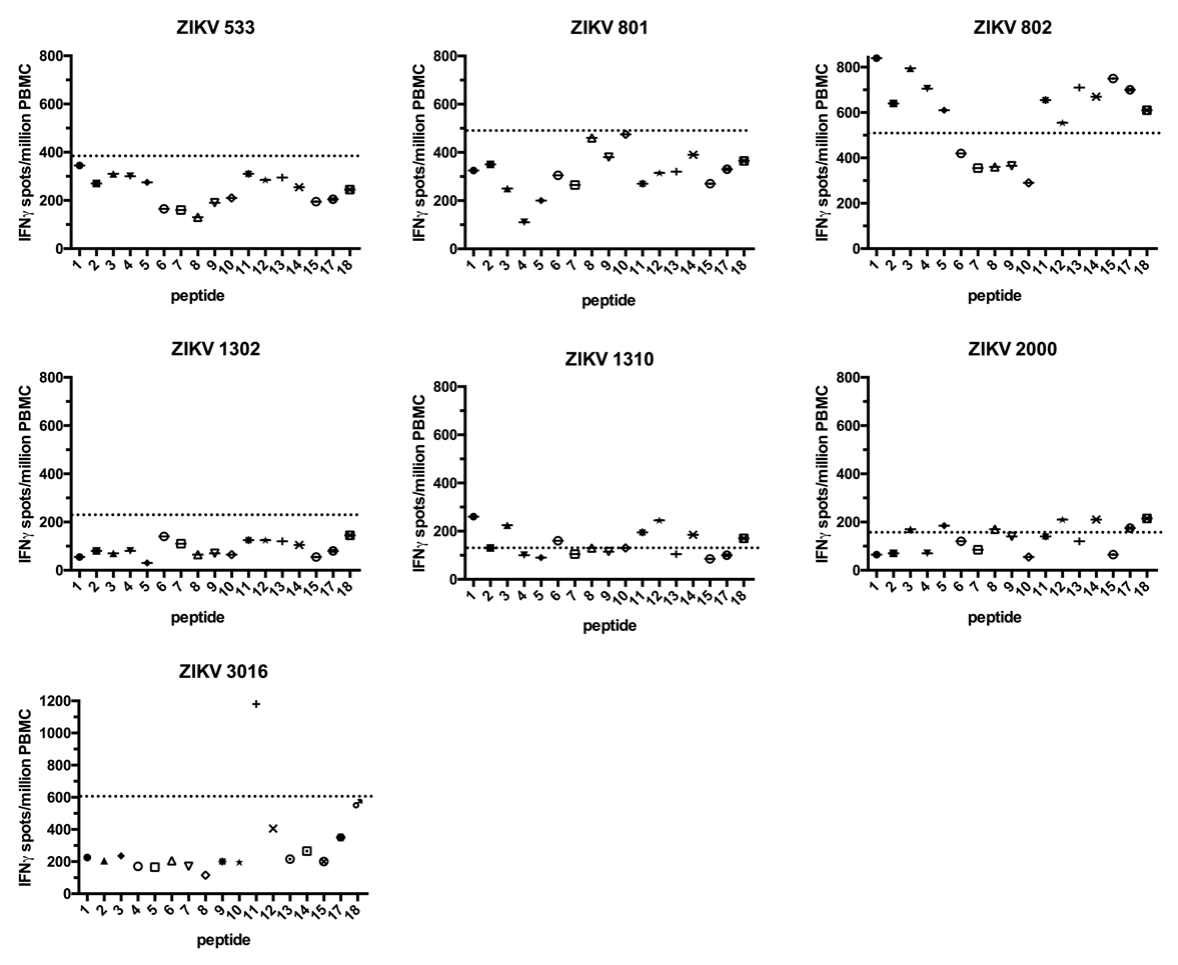
**Identification of ZIKV Epitope.** PBMCs from ZIKV-infected subjects were stimulated with peptides 1-18 and IFNγ production was evaluated by ELISPOT. The dashed line represents the background level of IFNγ in the absence of stimulation multiplied by 2.

**Supplementary Figure 1.**
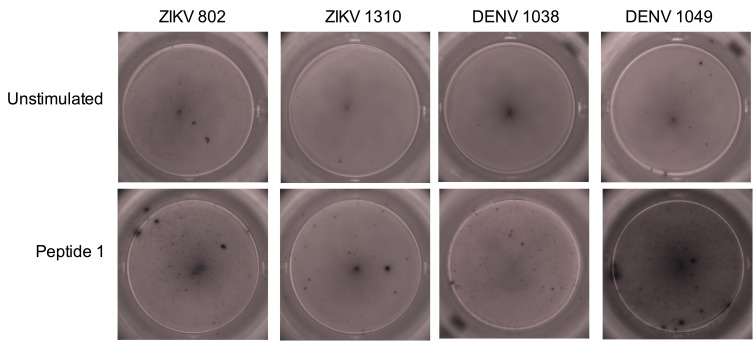
IFNgamma ELISPOT results for unstimulated PBMCs (top row) or stimulated with peptide 1 (lower row).

**Supplementary Figure 2.**
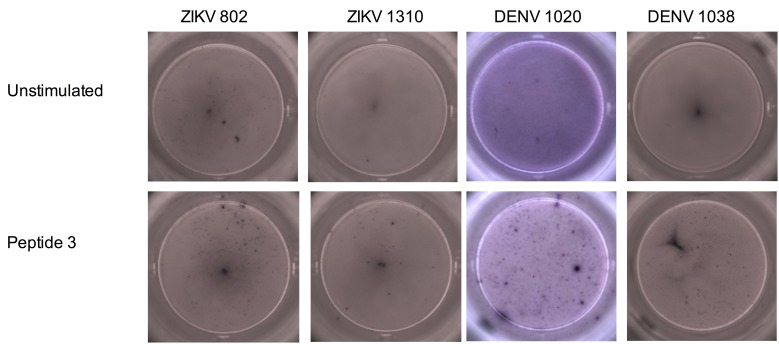
IFNgamma ELISPOT results for unstimulated PBMCs (top row) or stimulated with peptide 3 (lower row).

**Supplementary Figure 3.**
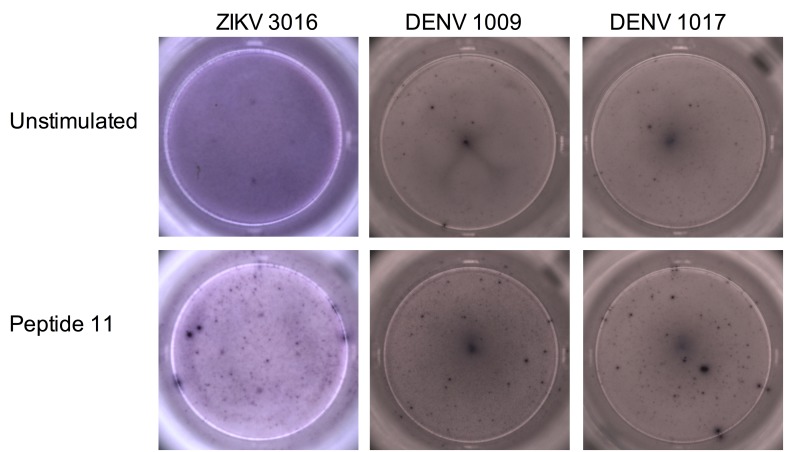
IFNgamma ELISPOT results for unstimulated PBMCs (top row) or stimulated with peptide 11 (lower row).

Next, we performed ELISPOTS with PBMCs obtained from 7 Brazilian patients with a previous ZIKV infection (Table 3). The patients denoted ZIKV 533, 801, 1302, and 2000 did not show IFNγ production above background for all peptides tested (Figure 2). Patient 3016 responded to peptide 11 only (2-fold over the background), and 802 and 1310 had a weak IFNγ response against many peptides including 1, 3, and 11 (less than 2-fold over the background, Figure 2 and Supplementary Figures 1, 2, and 3).

**Table 3.** Patient Demographics

Groups	Donor ID	Gender	Age	History of Dengue*	Past YF Immunization*	Day after the onset of symptoms or DENV-TV003 immunization of blood sample collection	Reported symptoms	PCR	
								Urine	Blood
ZIKV	801	F	53	yes (twice)	yes	19	Fever, myalgia	+	nd
	802	F	29	no	yes	19	Myalgia	+	nd
	533	F	56	yes	yes	159	Myalgia, abdominal pain	+	**-**
	1302	M	35	no	yes	109	Myalgia, nausea, abdominal pain	+	nd
	1310	F	32	no	no	12	Fever, myalgia, retro orbital pain	nd	+
	2000 **	F	37	no	yes	15	Fever, myalgia, skin rash	**-**	**-**
	3016	F	50	no	unk	16	Pruriginous skin rash, conjuctivitis, myalgia	+	+
DENY	1007	F	38	no	unk	90	Mild skin rash	na	na
	1009	M	32	no	unk	90	Mild skin rash	na	na
	1011	F	56	no	unk	90	none	na	na
	1016	M	47	no	unk	90	none	na	na
	1017	F	56	no	unk	90	none	na	na
	1020	F	45	no	unk	90	Mild skin rash	na	na
	1021	F	50	no	unk	90	none	na	na
	1038	F	35	no	unk	90	Mild skin rash	na	na
	1040	F	48	no	unk	90	Mild skin rash	na	na
	1049	F	37	no	yes	90	Mild skin rash	na	na

*Informed by the patient/participant, except for DENV-TV003 vaccinees (PRNT pre-vaccination was negative)

** Diagnosis based on symptoms

nd: Not done

na: Not applicable

unk: Unknown

YF: Yellow Fever

**Figure 3.**
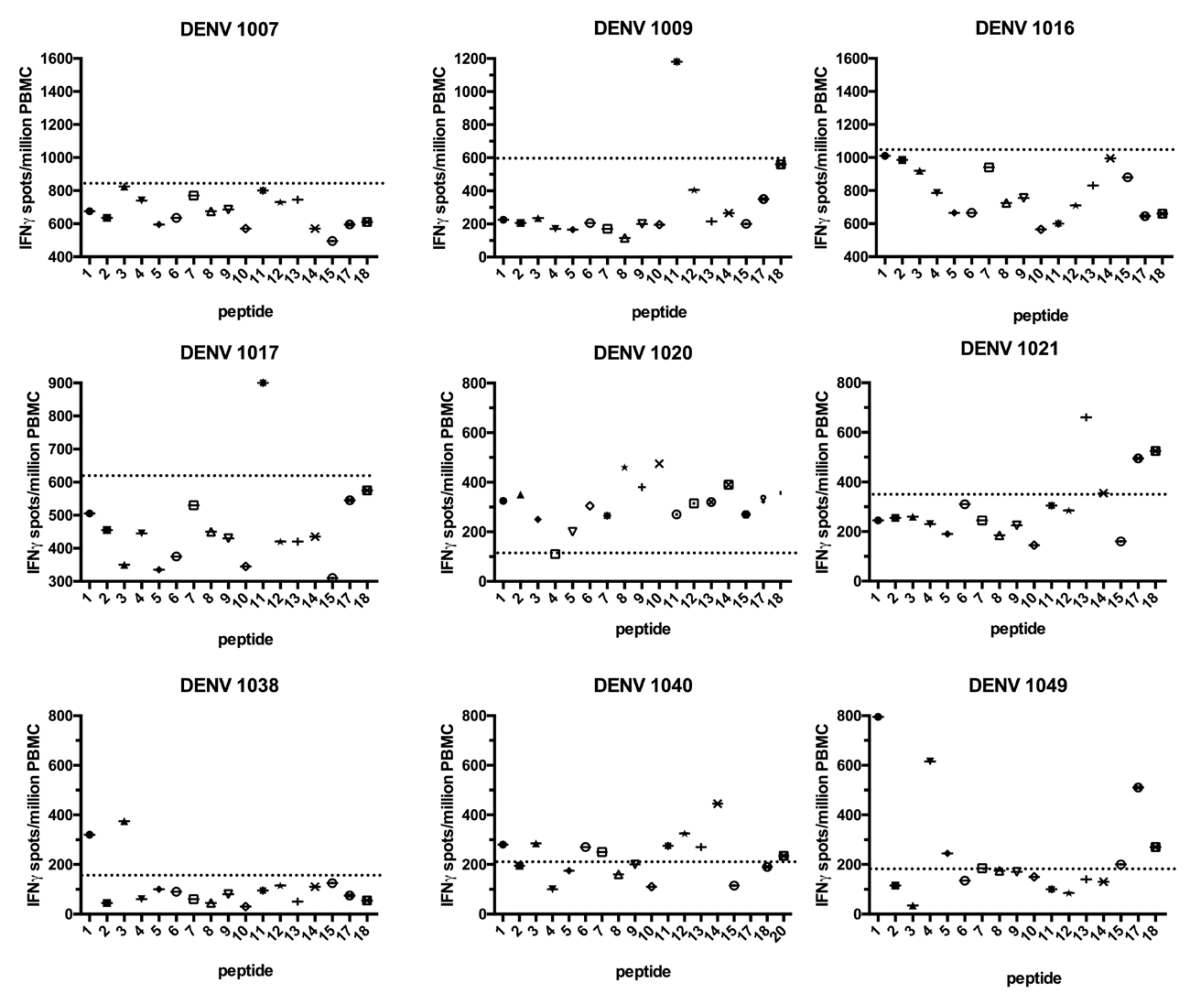
**Identification of DENV-ZIKV Cross-reactive Peptides.** PBMCs from DENV-vaccinated subjects were stimulated with peptides 1-18 and IFNγ production was evaluated by ELISPOT. The dashed line represents the background level of IFNγ in the absence of stimulation multiplied by 2.

We then stimulated PBMCs obtained from 9 Brazilian individuals, previously vaccinated against DENV (Tables 3 and 4), with the ZIKV peptides. These DENV-vaccinated individuals received the live, attenuated tetravalent vaccine TV003 [31] as part of a phase 2 clinical trial (clinicaltrials.gov identifier: NCT01696422) currently being conducted in Brazil, and samples were collected before ZIKV arrived in São Paulo. Two of the DENV-vaccinated participants (1007 and 1016) did not respond to any of the peptides, and 2 responded only to peptide 11 (1009 and 1017) (Figure 3 and Supplementary Figures 1 and 2). The remaining 5 DENV-vaccinated individuals (1021, 1020, 1038, 1040, and 1049) responded to at least 2 of the peptides, albeit some of the responses were modest (Figure 3 and Supplementary Figures 1 and 3). Overall, peptides 1, 3, and 11 induced a response in both ZIKV-infected and DENV-vaccinated individuals, with the strongest responses observed for peptides 1 and 11. The different time points at which samples were collected made a direct comparison of the magnitude of the IFNγ response between the ZIKV-infected patients and the DENV-vaccinated individuals impossible. Some had high background IFNγ levels that could have masked weak antigen-specific responses. However, we could still detect antigen-specific responses in those participants (ie, ZIKV 3016, DENV 1009, and DENV 1017).

**Table 4:** HLA-I alleles of DENV-vaccinated participants.

Samples	HLAA	HLAA	HLAB	HLAB	HLAC	HLAC
DENV 1007	A*03:ABWKR	A*29:ABWMY	B*44:ABYSJ	B*58:ACBCJ	C*03:ACPXG	C*16:ACPWC
DENV 1009	A*24:ACPYT	A*24:HYYJ	B*40:ACKGC	B*51:ABUUP	C*03:ABUVB	C*14:ABUWF
DENV 1011	A*02:ZRVU	A*02:ZRVU	B*41:ZRNP	B*50:ABWUY	C*06:ABWZK	C*07:ABWZU
DENV 1016	A*02:ABWJM	A*24:ACAEP	B*44:ABYRU	B*44:ABWUJ	C*05:ACBEA	C*16:ABZVM
DENV 1017	A*29:ABWMX	A*31:ABWNG	B*39:VZGY	B*44:ACBUY	C*07:ABXAG	C*16:ABXBJ
DENV 1020	A*02:ABVHV	A*23:ZKEV	B*27:ACMMT	B*58:ACJEC	C*05:ABZTT	C*07:ABZUE
DENV 1021	A*29:ACDMP	A*29:ACDMP	B*14:ABYJF	B*44:ABZPN	C*08:ACAMF	C*16:ABZVM
DENV 1038	A*02:ABZCH	A*33:ABYFZ	B*15:KPAE	B*44:ACNAV	C*03:ACFEZ	C*14:ACFHD
DENV 1040	A*02:ABUKX	A*29:ABUKV	B*38:WDAZ	B*44:ABZAB	C*12:ABZAC	C*16:ABZAD
DENV 1049	A*02:ABVRS	A*26:ABVSS	B*14:ABWRH	B*51:ABWXE	C*08:ACBFG	C*15:ACBGC

**Figure 4.**
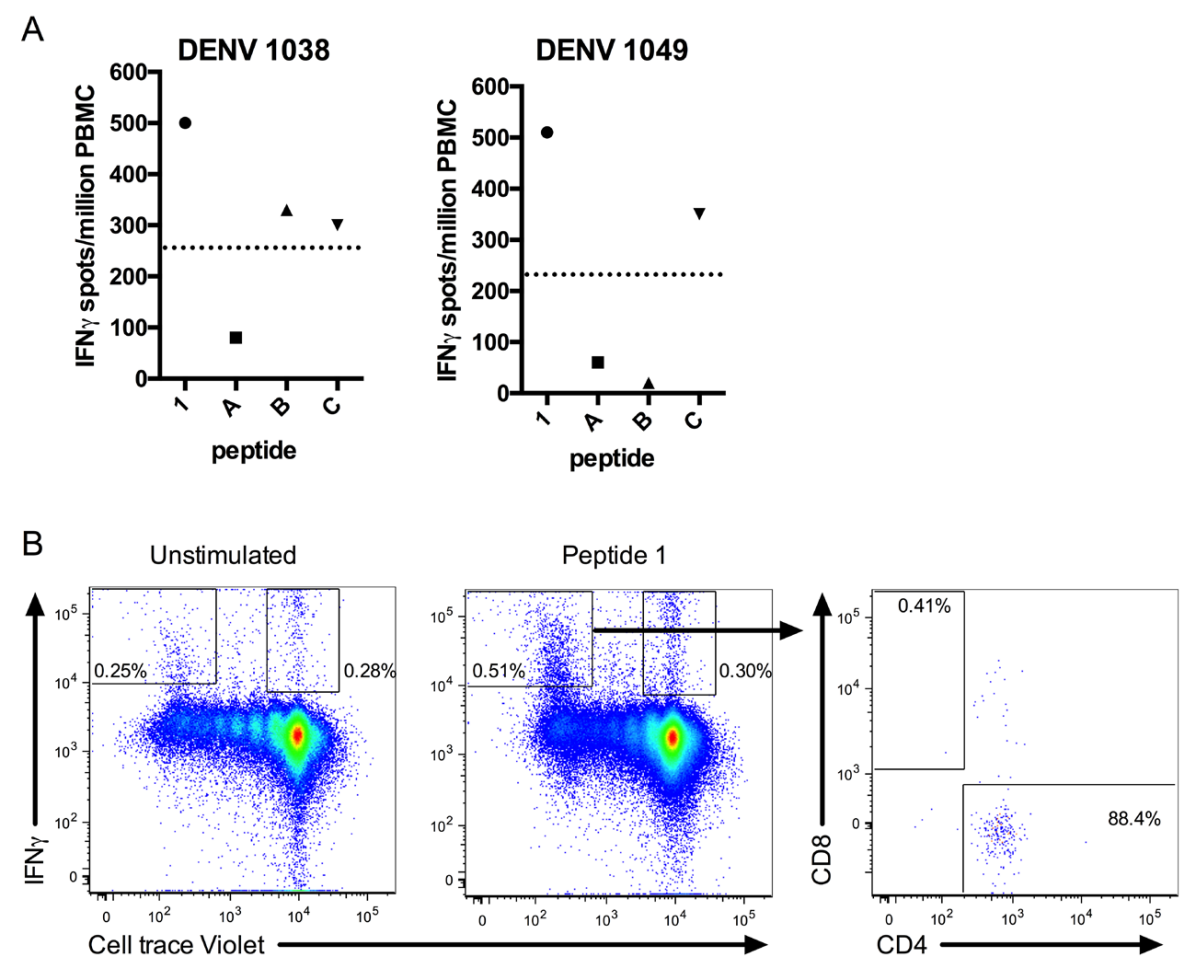
**Fine Mapping of Peptide 1.** IFNγ spots per million PBMCs after stimulation with peptides 1, A, B, and C for DENV 1038 and 1049. The dashed line represents the background level of IFNγ in the absence of stimulation multiplied by 2 (A). CellTrace Violet labeled PBMCs were stimulated for 6 days with peptide 1 in the presence of IL-2 (20 U/ml) before re-stimulation with peptide 1 for 12 hours in the presence of monensin. As a negative control, PBMCs were cultured with IL-2 in absence of peptide stimulation. Cells were then stained for CD3, CD4, CD8, and IFNγ. Representative flow plots (B).

Finally, we selected peptide 1 to use for fine epitope mapping with PBMCs from participants DENV 1038 and DENV 1049, who were chosen based on their strong responses to this peptide and because they carry the predicted HLA-I allele for this peptide (HLA-A2, Tables 2 and 4). The PBMCs were stimulated with the complete 15-mer peptide or 3 overlapping 9-mer peptides corresponding to the first 9 (A), the middle 9 (B), and the last 9 (C) amino acids of peptide 1 (Table 2). Peptide A was predicted to bind strongly to HLA-A2, and both DENV 1038 and DENV 1049 carried this allele. Stimulation with the complete peptide resulted in an IFNγ response, but surprisingly, stimulation with peptide A did not induce a response (Figure 4 A). Similarly, stimulation with peptides B and C resulted in no or weak IFNγ responses. The requirement for the full 15-mer peptide suggests that peptide 1 might be presented by HLA-II. To test this, flow cytometry was used to determine if peptide 1 generated a CD4+ or CD8+ T-cell response. The PBMCs from participants DENV 1038 and DENV 1049 were labeled with CellTrace Violet before being cultured for 6 days in the presence or absence of the peptide. The cells were then re-stimulated with the peptide, and antigen-specific cells were identified as CellTrace Violet low and IFNγ+. Background IFNγ production (CellTrace Violet high, IFNγ+) did not change after peptide stimulation, but an antigen- specific response was identified for both donors (Figure 4 B). The majority of the CellTrace Violet low and IFNγ+ cells were CD4+ T cells for both DENV-vaccinated participants. We then determined the HLA-II alleles carried by DENV 1038 (HLA-DRB1*04 and HLADRB1*13) and DENV 1049 (HLA-DRB1*03 and HLA-DRB1*04) and used the IEDB analysis resource consensus tool [24, 25] to evaluate if peptide 1 could be presented by these alleles. We found that peptide 1 is predicted to be a strong binder for HLA-DRB1*03 (percentile rank 3.22) and HLA-DRB1*13 (percentile rank 2.40). While we only observed a CD4+ T-cell response for subjects DENV 1038 and DENV 1049, it is still possible that other DENV-vaccinated individuals carrying HLA-A2 will generate a CD8+ T-cell response against peptide 1. Because of sample limitations, we could not perform fine epitope mapping for the ZIKV-infected patients. Further studies will be needed to determine the HLA restriction of the other ZIKV epitopes identified.

T-cell cross-reactivity could have a positive or negative impact on ZIKV infection. In fact, many ZIKV-infected people will have had a prior DENV infection or been exposed to other flaviviruses, such as those receiving the YF vaccine. Prior infections or vaccinations sometimes negatively impact the response to a subsequent infection, a phenomenon that has been called “original antigenic sin (OAS)”. This OAS occurs in serial infections with pathogens that share cross-reactive antigens. For example, preformed memory T cells, which cross-react with low avidity to epitopes presented in subsequent infections, could dampen the response of high-avidity T cells [32–34]. Alternatively, cross-reactive T cells might have a protective effect. This is supported by evidence showing that flavivirus-immune individuals have better protection than flavivirus-naive subjects following DENV vaccination [35]; in addition, T-cell cross-reactivity is further complicated by the highly polymorphic nature of HLA molecules. In other words, prior DENV infection or vacci-nation could impact the ZIKV T-cell response. In mice, ZIKV/DENV cross-reactive CD8+ T cells induced by DENV infection were found to contribute to protection against ZIKV infection [22].

In this study, we have identified ZIKV T-cell epitopes and demonstrated that vaccination against DENV induces a T-cell response against ZIKV. Given the similarity in the T-cell response between DENV natural infection and vaccination [36], it is likely that natural DENV infection can also generate a T-cell response against ZIKV. More research is needed to determine if this cross-reactive immune response is protective or contributory to ZIKV pathology. In particular, the ZIKV CD4+ T-cell response induced by DENV vaccination and identified in this study could contribute to the appearance of cross-reactive antibodies mediating ADE. A better understanding of the interactions between ZIKV and DENV immunity, whether induced by vaccination or by natural infection, will be critical to obtain safe and efficient vaccines.

## LIST OF ABBREVIATIONS

ZIKV: Zika virus
YF: Yellow Fever
DENV: Dengue virus
ADE: Antibody dependent enhancement
OAS: Original antigenic sin
nd: Not done
na: Not applicable
unk: Unknown
